# Circular RNA circ_0004277 Inhibits Acute Myeloid Leukemia Progression Through MicroRNA-134-5p / Single stranded DNA binding protein 2

**DOI:** 10.1080/21655979.2022.2059609

**Published:** 2022-04-12

**Authors:** Yao Liu, Xi Chen, Jingyang Liu, Yinglan Jin, Wei Wang

**Affiliations:** a Department of Hematology, The Second Affiliated Hospital of Harbin Medical University, Harbin, Heilongjiang Province, China; b Department of Gastroenterology, The Second Affiliated Hospital of Harbin Medical University, Harbin, Heilongjiang Province, China

**Keywords:** Circ_0004277, miR-134-5p, acute myeloid leukemia, proliferation

## Abstract

Circular RNAs (circRNAs) are crucial non-coding RNAs in the process of tumorigenesis. Nevertheless, the biological function of circ_0004277 in acute myeloid leukemia (AML) is blurred. Microarray data of circRNAs were utilized to evaluate circRNAs’ differential expression in AML. Quantitative real-time polymerase chain reaction (qRT-PCR) was executed to determine circ_0004277 and microRNA-134-5p (miR-134-5p) expression levels. The growth, migration and invasion of AML cells were tested by the cell counting kit-8 and Transwell experiment. Dual-luciferase reporter gene experiment, RNA immunoprecipitation (RIP) experiment and RNA pull-down experiment were executed to determine the targeting relationship between circ_0004277 and miR-134-5p. Western blot assay was used to detect single stranded DNA binding protein 2 (SSBP2) expression. We observed that circ_0004277 was down-regulated in AML, while miR-134-5p was up-regulated. Functionally, circ_0004277 overexpression or inhibition of miR-134-5p remarkably suppressed AML cell viability, migration and invasion. Furthermore, miR-134-5p served as a direct downstream target of circ_0004277 and SSBP2 was identified as a target of miR-134-5p. Compensation experiments showed that miR-134-5p mimics abolished the biological function of circ_0004277 on malignant phenotypes of AML cells. Collectively, circ_0004277 impedes AML development by adsorbing miR-134-5p and up-regulating SSBP2.

## Graphical Abstract

**Figure:**
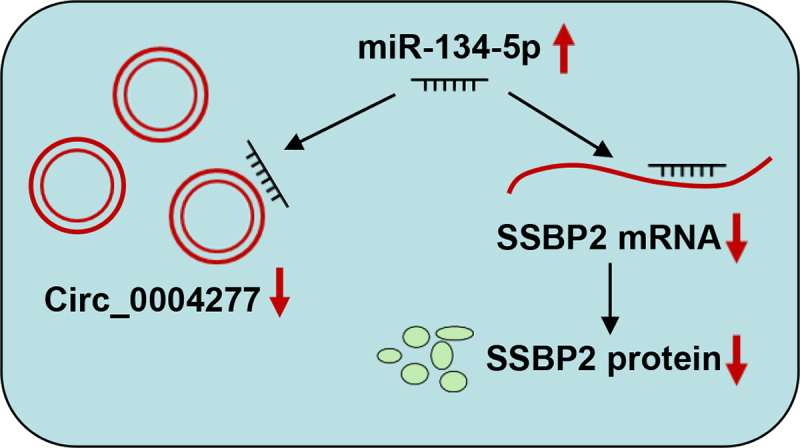

## 1. Introduction

Acute myeloid leukemia (AML) is featured by a series of immature myeloid progenitor cells that proliferate malignantly [1]. These cells entirely or partly disable to differentiate and accumulate in the bone marrow, peripheral blood and other organs [1]. Reportedly, there are about more than 19,520 new cases of AML and 10,670 deaths/year in the United States [2]. Despite there are treatment options such as chemotherapy, bone marrow transplantation and targeted therapies, AML patients still have a high mortality [3]. Hence, it is imperative to probe the possible mechanisms implicated in AML progression, which may improve the discovery of innovative diagnostic molecular biomarkers and effective therapeutic targets to promote the survival of AML patients.

Circular RNAs (circRNAs) are generated by ‘reverse splicing’ of pre-mRNA transcripts, with a covalently closed circular structure [4]. Multiple circRNAs are considered as potential biomarkers for several cancers [5–9]. For example, circ-LDLRAD3 is highly expressed in pancreatic cancer tissues, and its overexpression is closely associated with vascular invasion and metastasis [7]. Circ_0000745 works as a tumor-promoting factor in cervical cancer, and it enhances the growth, migration and invasion of cancer cells by suppressing E-cadherin expression [8]. Interestingly, circ_0000745 is lowly expressed in gastric cancer, and its expression correlates with the degree of tumor differentiation [9]. In this work, analysis of the data from the circRNA microarray (GSE94591 dataset) reveals that circ_0004277 is markedly down-regulated in AML. Nonetheless, the biological function of circ_0004277 in AML is blurred.

MicroRNAs (miRNAs) are highly conserved single-chain non-coding RNAs, approximately 11–22 *nt* in length [10]. Up to now, thousands of miRNAs are recognized in mammals [11,12]. Many miRNAs are reported to be potential biomarkers or therapeutic targets for different diseases [13,14].

As mentioned above, differentially expressed circRNAs in AML were screened using the data from Gene Expression Omnibus (GEO) database, and it is revealed that circ_0004277 is probably down-regulated in the progression of AML. This study aimed to probe the possible biological functions and underlying mechanisms of circ_0004277 in AML cells, and with bioinformatics analysis, we hypothesized that circ_0004277 might participate in AML progression via inhibiting miR-134-5p and up-regulating single stranded DNA binding protein 2 (SSBP2).

## 2. Materials and methods

### 2.1. Clinical data

Bone marrow specimens were collected from 37 AML patients and 37 healthy donors. Thirty-seven patients with AML were newly diagnosed according to French-American-British classification and WHO criteria. All subjects did not receive any treatment before sample collection. This research was endorsed by the Committee of the Second Affiliated Hospital of Harbin Medical University (201,904,003), conducted following the Declaration of Helsinki, and informed consent was available from each subject [15].

### 2.2. Cell culture

Human bone marrow stromal cell line HS-5 and human AML cell lines (KG-1a, THP-1, K562 and U937) were procured from the American Type Culture Collection (Manassas, VA, USA). Cells were cultured in Roswell Park Memorial Institute-1640 (RPMI-1640, Gibco, Rockville, MD, USA) containing 10% fetal bovine serum (FBS) (Gibco, Rockville, MD, USA) at 37°C with 5% CO_2_ [15].

### 2.3. Quantitative real-time polymerase chain reaction (qRT-PCR)

Total RNA was collected using TRIzol reagent (Invitrogen, Carlsbad, CA, USA) according to the manufacturer’s instruction. Reverse transcription was performed with a PrimeScript RT Reagent Kit (Takara, Dalian, China), and qRT-PCR was executed using the Universal SYBR Green Master Mix kit (Takara, Dalian, China). The divergent primer for circ_0004277 was designed by Geneseed (Guangzhou, China), and the other primers were designed and synthesized by RiboBio (Guangzhou, China) and Sangon Biotech (Shanghai, China). The sequences of the primers utilized for qRT-PCR are listed in Table 1. 2^−ΔΔCt^ method was utilized to analyze the relative expression. For RNase treatment assay, 2 μg of total RNA was incubated with or without 3 U/mg RNase R for 30 minutes at 37°C, and then qRT-PCR was performed [15].

**Table 1.** Primer sequence used in this study

Gene	Sequence
circ_0004277	F: 5’-AACAAAAGCCAGTCACAGCA-3’ R: 5’-CATCAATCGCTTGTCCTTCA-3’
miR-134-5p	F: 5’-GAAGCTCATTGGAGACCCTAAC-3’ R: 5’-CAACCTCTAAGTCGTGCTCATAC-3’
SSBP2	F: 5’-GAAGTCAGCCATTACTCCCCA-3’ R: 5’-CTGCATTGGCCCACCCATATT-3’
GAPDH	F: 5’-TGGTCACCAGGGCTGCTT-3’ R: 5’-AGCTTCCCGTTCTCAGCC-3’
U6	F: 5’-CTCGCTTCGGCAGCACA-3’ R: 5’-AACGCTTCACGAATTTGCGT-3’

F, forward; R, reverse.

### 2.4. Cell transfection

Human circ_0004277 overexpression vector (oe-circ_0004277) and the empty plasmid (oe-NC), miR-134-5p mimics, miR-134-5p inhibitors and negative control miRNA were procured from RiboBio Co., Ltd. (Guangzhou, China). Human circ_0004277 transcript cDNA was inserted into the pcDNA3.1(+) (VT1001, YouBio, Chongqing, China) to construct the overexpression plasmid. They were transfected into U937 and KG-1a cells with Lipofectamine® 2000 (Invitrogen, Carlsbad, CA, USA) according to the manufacturer’s instruction [15].

### 2.5. Cell counting kit-8 (CCK-8) assay

Both U937 and KG-1a cells were plated in 96-well plates (1000 cells/well) containing 100 µL of medium and cultured at 37°C with 5% CO_2_. Ten microliters of CCK-8 solution (Dojindo, Kumamoto, Japan) was supplemented into each well at the specific time points (0, 1, 2, 3 and 4 days). After 2 hours, the absorbance of the cells in each well was measured using a Model 680 Microplate Reader (Bio-Rad, Richmond, CA, USA) at a wavelength of 450 nm [15].

### 2.6. Transwell experiment

Cell migration and invasion were determined by the Transwell experiment using a 24-well Transwell system (Corning, Corning, NY, USA). The membrane of the Transwell system was paved with Matrigel (BD Bioscience, San Jose, CA, USA) for the invasive experiment. U937 and KG-1a cells (1 × 10^4^ cells/well) cultured in RPMI-1640 medium without FBS were inoculated into the top compartment, while RPMI-1640 medium containing 10% FBS was injected into the bottom compartment. The cells on the bottom side of the membrane were fixed with 95% methanol after 24 hours and stained for 15 minutes with 0.5% crystal violet solution (Sigma, St. Louis, MO, USA). Finally, the cells were counted under a microscope in three random fields of view [16]. In the migration experiment, the procedures are the same, except that the Matrigel was not used.

### 2.7. Luciferase reporter gene assay

Circ_0004277 or SSBP2 sequence containing miR-134-5p binding site was amplified and inserted into pGL3 basic reporter vectors (Promega, Madison, WI, USA) to establish the circ_0004277-wild-type (WT) or SSBP2-WT reporter vector. Meanwhile, the mutated circ_0004277 or SSBP2 sequence was inserted into the pGL3 basic reporter vectors to establish the circ_0004277-mutant (MUT) or SSBP2-MUT reporter vector. Circ_0004277-WT or circ_0004277-MUT reporter vector was co-transfected with miR-134-5p mimic or control miRNA into U937 and KG-1a cells. SSBP2-WT or SSBP2-MUT reporter vector was co-transfected with miR-134-5p mimic or control miRNA into U937 and KG-1a cells. Ultimately, the relative luciferase activity of each group was measured by a Dual-Luciferase Assay System (Promega, Madison, WI, USA) [16].

### 2.8. RNA-binding protein immunoprecipitation (RIP) experiment

The EZMagna RIP Kit (Millipore, Billerica, MA, USA) was utilized to validate the interaction between miR-134-5p and circ_0004277 according to the manufacturer’s instruction. Cell extract was incubated with magnetic beads conjugated with anti-Ago2 or IgG antibody (Millipore, Billerica, MA, USA). The magnetic beads were rinsed, and then the complex was incubated with Proteinase K to eliminate proteins. Ultimately, the RNA was isolated from the immunoprecipitate, and qRT-PCR was performed [16].

### 2.9. RNA pull-down experiment

Biotinylated miR-134-5p, mutated miR-134-5p, or control mimics were transfected into U937 and KG-1a cells, respectively. The cells were harvested after transfection and lysed with lysis buffer (Ambion, Austin, Texas, USA). Based on the manufacturer’s instruction, the cell lysate was incubated with Dynabeads M-280 Streptavidin (Invitrogen, Carlsbad, CA, USA). The beads were rinsed twice with pre-chilled lysis buffer, three times with low salt buffer, and once with high salt buffer after being incubated at 4°C for 3 hours. Subsequently, the immunoprecipitated RNA was isolated and purified, and finally qRT-PCR was conducted to detect the enrichment of circ_0004277 [16].

### 2.10. Western blot assay

RIPA lysis buffer (Beyotime, Shanghai, China) was used to extract total protein from the cells, and the protein was quantified using a BCA kit (Beyotime, Shanghai, China). Protein samples were then separated by sodium dodecyl sulfate polyacrylamide gel electrophoresis and transferred onto PVDF membranes. Then, the membranes were blocked with 5% defatted milk, and washed with tris buffered saline tween, and then incubated with anti-SSBP2 antibody (1:1,000; Thermo Fisher Scientific, Waltham, MA, USA) at 4°C overnight, then incubated with HRP-conjugated anti-rabbit antibody (1:20,000; Thermo Fisher Scientific, Waltham, MA, USA) at room temperature for 1 h. The BeyoECL Plus kit (Beyotime, Shanghai, China) was used to develop the protein bands. Image J software was applied to determine the gray values of the protein bands [16].

### 2.11. Statistical analysis

The data in the present study were expressed as the ‘mean ± standard deviation’ of three independent assays. GraphPad Prism 8.0 (GraphPad Software, Inc., San Diego, CA, USA) and SPSS 17.0 software (SPSS Inc., Chicago, IL, USA) were employed for graphing and statistical analysis, respectively. Comparisons between two and multiple groups were conducted with student’s *t*-test and one-way ANOVA, respectively. Pearson’s correlation coefficient was employed for correlation analysis. *P* < 0.05 implied statistical significance.

## 3. Results

In this study, we explored the expression characteristics, biological function and underlying mechanism of circ_0004277 in AML. We found that circ_0004277 expression level was decreased in AML, and it suppressed the progression of AML via miR-134-5p/SSBP2 axis. The study provided a novel insight for AML therapy.

### 3.1. Circ_0004277 expression is reduced in AML

**Figure 1.**
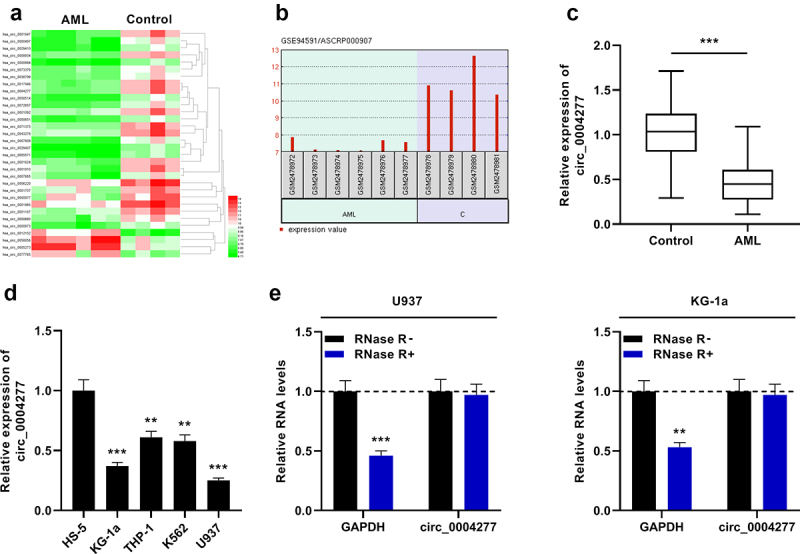
Circ_0004277 expression is remarkably up-regulated in AML. A. Heat map showed the differentially expressed circRNAs (bone marrow mononuclear cells of AML patients v.s. bone marrow mononuclear cells of healthy controls, GSE94591), and the filter criteria was *P* < 0.05,Log2(Change fold)> 2.5. B. The expression of circ_0004277 in bone marrow mononuclear cells of AML patients was significantly down-regulated compared with that of healthy controls (GSE94591). C. qRT-PCR showed that circ_0004277 expression was lower in the bone marrow samples of 37 AML patients than that in 37 healthy controls. D. qRT-PCR revealed that circ_0004277 expression in AML cells (KG-1a, THP-1, K562 and U937) was down-regulated compared with the human normal stromal cells (HS-5). E. qRT-PCR showed that circ_0004277 was resistant to RNase R. ** *P* < 0.01, and *** *P* < 0.001.

In this work, first of all, we analyzed the data of GSE94591 dataset and visualized the differentially expressed circRNAs in bone marrow mononuclear cells of AML patients and healthy controls by the GEO2R online tool. The significantly aberrantly expressed circRNAs were showed in the heat map (*P* < 0.05, Log2(Change fold)>2.5, Figure 1(a)). As shown in Figure 1(b), circ_0004277 was remarkably down-regulated in AML samples (logFC = −3.7637523, *P* = 0.00000141). Moreover, the data of qRT-PCR showed that circ_0004277 expression was markedly lower in bone marrow specimens of 37 AML patients than in the control group (Figure 1(c)). Furthermore, qRT-PCR showed that circ_0004277 was markedly under-expressed in AML cells (KG-1a, THP-1, K562 and U937) relative to the human normal bone marrow stromal cells (HS-5) (Figure 1(d)). Besides, in both U937 and KG-1a cells, the level of GAPDH was remarkably weakened after RNase R treatment, while circ_0004277 was observed to be resistant to RNase R (Figure 1(e)). The findings implied that circ_0004277 had a circular structure and may participate in AML development.

### 3.2. Circ_0004277 restrains the viability, migration and invasion of U937 and KG-1a cells

**Figure 2.**
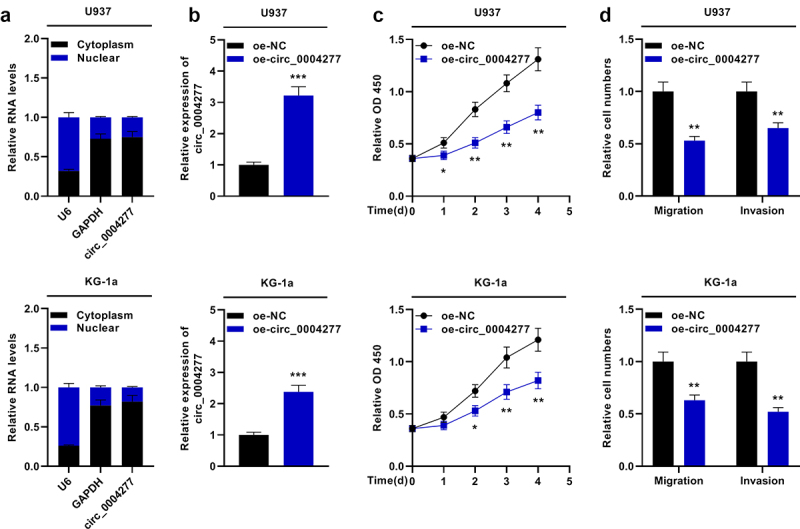
Circ_0004277 impedes the proliferation and metastasis of U937 and KG-1a cells. A. qRT-PCR showed that circ_0004277 was mainly located in the cytoplasmic fractions of U937 and KG-1a cells. GAPDH and U6 were considered as the markers of cytoplasm and nucleus. B. qRT-PCR showed circ_0004277 expression was higher in the circ_0004277 overexpression plasmid group compared with the empty plasmid group. C. CCK-8 assay revealed that the viability of U937 and KG-1a cells transfected with circ_0004277 overexpression plasmid was suppressed, than that of U937 and KG-1a cells transfected with the empty plasmid. D. Transwell assay showed that the number of migratory and invasive cells were less in U937 and KG-1a cells transfected with circ_0004277 overexpression plasmid than that of U937 and KG-1a cells transfected with the empty plasmid. * *P* < 0.05, ** *P* < 0.01, and *** *P* < 0.001.

To probe the biological function of circ_0004277 in AML development, two cell lines (U937 and KG-1a cells) with lowest circ_0004277 expression were selected. The nuclear-cytoplasm separation assay verified that circ_0004277 was predominantly situated in the cytoplasm (Figure 2(a)). Moreover, circ_0004277 overexpression plasmid and empty plasmids were transfected into U937 and KG-1a cells to construct the circ_0004277 overexpression model (Figure 2(b)). The biological function of circ_0004277 on AML cell viability, migration and invasion was then explored. CCK-8 assay showed that circ_0004277 overexpression suppressed the growth of U937 and KG-1a cells (Figure 2(c)). Additionally, the Transwell assay suggested that circ_0004277 overexpression remarkably repressed the migration and invasion of U937 and KG-1a cells (Figure 2(d)). These experiments indicated that circ_0004277 was a tumor-suppressor in AML progression.

### 3.3. miR-134-5p is a direct downstream target of circ_0004277

**Figure 3.**
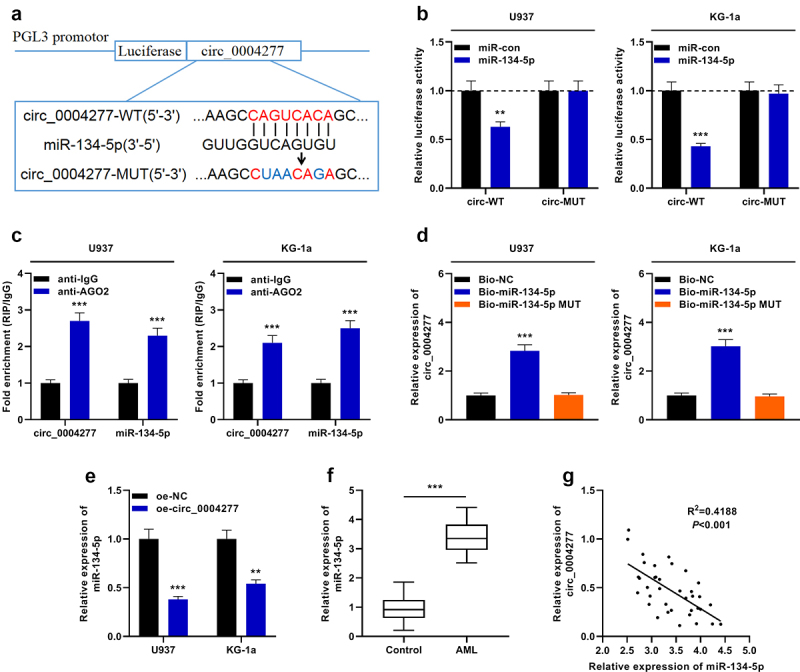
Circ_0004277 directly targets miR-134-5p. A. Bioinformatics was employed to predict the binding site between circ_0004277 and miR-134-5p. B. Dual-luciferase reporter gene assay assays demonstrated that miR-134-5p could negatively regulate the luciferase activity of circ_0004277-WT, rather than circ_0004277-MUT in U937 and KG-1a cells. C. RIP assay indicated that circ_0004277 and miR-134-5p were enriched in anti-Ago2 group, suggesting circ_0004277 and miR-134-5p were directly interacted. D. RNA pull-down assay showed that circ_0004277 and miR-134-5p could combine with each other. E. qRT-PCR indicated that miR-134-5p expression was decreased in the circ_0004277 overexpression plasmid group compared with the empty plasmid group. F. qRT-PCR indicated that miR-134-5p expression was increased in the bone marrow samples of 37 AML patients compared with that of 37 healthy donors. G. circ_0004277 expression in AML tissues was negatively correlated with miR-134-5p expression in bone marrow samples of the patients with AML. ** *P* < 0.01, and *** *P* < 0.001.

To decipher the downstream mechanism of circ_0004277 in AML development, the bioinformatics tool was utilized to predict the possible target miRNAs of circ_0004277, and miR-134-5p was predicted to be a candidate target of circ_0004277 (Figure 3(a)). Dual-luciferase reporter gene assay showed that miR-134-5p suppressed the luciferase activity of the circ_0004277-WT reporter, while it had no effect on luciferase activity in the circ_0004277-MUT reporter, indicating that circ_0004277 can bind directly to miR-134-5p (Figure 3(b)). Furthermore, the data of the RIP experiment and RNA pull-down experiment suggested that circ_0004277 directly interacted with miR-134-5p in U937 and KG-1a cells (Figure 3(c,d)). Additionally, qRT-PCR experiment revealed a remarkable decrease of miR-134-5p expression in the circ_0004277 overexpression group (Figure 3(e)). Besides, miR-134-5p was markedly up-regulated in AML samples of the patients (figure 3(f)). Besides, Pearson correlation analysis showed that circ_0004277 was negatively correlated with miR-134-5p expression in AML samples (R^2^ = 0.4188, *P* < 0.001) (Figure 3(g)).

### 3.4. Inhibition of miR-134-5p suppresses the growth, migration and invasion of U937 and KG-1a cells

**Figure 4.**
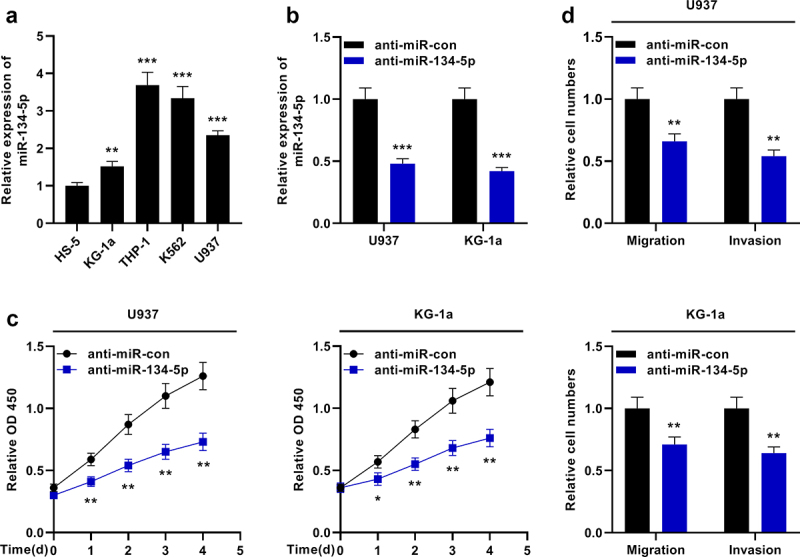
MiR-134-5p inhibits the proliferation and aggressiveness of KG-1a and U937 cells. A. qRT-PCR showed that miR-134-5p expression was up-regulated in AML cell lines (KG-1a, THP-1, K562 and U937) compared with the human normal stromal cells (HS-5). B. qRT-PCR revealed that miR-134-5p expression was down-regulated in U937 and KG-1a cells transfected with miR-134-5p inhibitor. C. CCK-8 assay showed the viability of KG-1a and U937 cells was significantly decreased after the transfection of anti-miR-134-5p. D. Transwell assay indicated that the migration and invasion of KG-1a and U937 cells were decreased after the transfection of anti-miR-134-5p. * *P* < 0.05, ** *P* < 0.01, and *** *P* < 0.001.

MiR-134-5p expression in AML cells was then investigated. qRT-PCR showed that miR-134-5p was remarkably overexpressed in AML cell lines compared with HS-5 cells (Figure 4(a)). MiR-134-5p inhibitors were transfected into U937 and KG-1a cells (Figure 4(b)). The effects of miR-134-5p on the growth, migration and invasion of AML cells were examined by the CCK-8 experiment and Transwell experiment, respectively, and it was revealed that the inhibition of miR-134-5p suppressed the growth and aggressiveness of AML cells (Figure 4(c,d)).

### 3.5. Circ_0004277 modulates the biological behaviors of AML cells through adsorbing miR-134-5p

**Figure 5.**
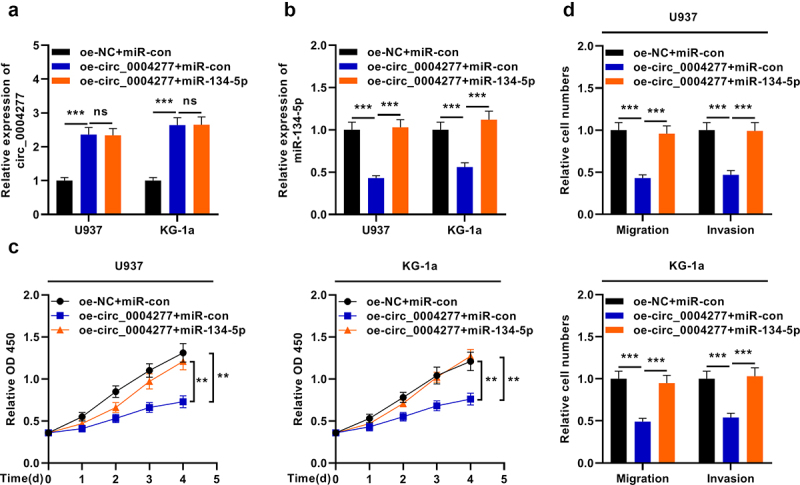
Circ_0004277 modulates the biological behaviors of AML cells by sponging miR-134-5p. A. qRT-PCR showed that the miR-134-5p mimics have no effect on the expression of circ_0004277. B. qRT-PCR indicated that miR-134-5p mimics could up-regulate the expression of miR-134-5p. C. CCK-8 assay showed that miR-134-5p mimics could reverse the effect of circ_0004277 overexpression plasmid on the viability of U937 and KG-1a cells. D. Transwell assay showed that the migration and invasion of U937 and KG-1a cells transfected with circ_0004277 overexpression plasmid was reversed by miR-134-5p mimics. ** *P* < 0.01, *** *P* < 0.001.

To further expound the mechanism of circ_0004277 in AML, three different transfection groups were established: oe-NC + miR-con, oe-circ_0004277 + miR-con and oe-circ_0004277 + miR-134-5p. qRT-PCR was used to detect circ_0004277 and miR-134-5p expression levels in the cells and it showed that the transfection was successful (Figure 5(a,b)). It was observed that the restoration of miR-134-5p reversed the effects of circ_0004277 overexpression on the viability, migration and invasion of AML cells (Figure 5(c,d)). Taken together, circ_0004277 may participate in AML progression through repressing miR-134-5p.

### 3.6. Circ_0004277 modulates the expression of SSBP2 through adsorbing miR-134-5p

**Figure 6.**
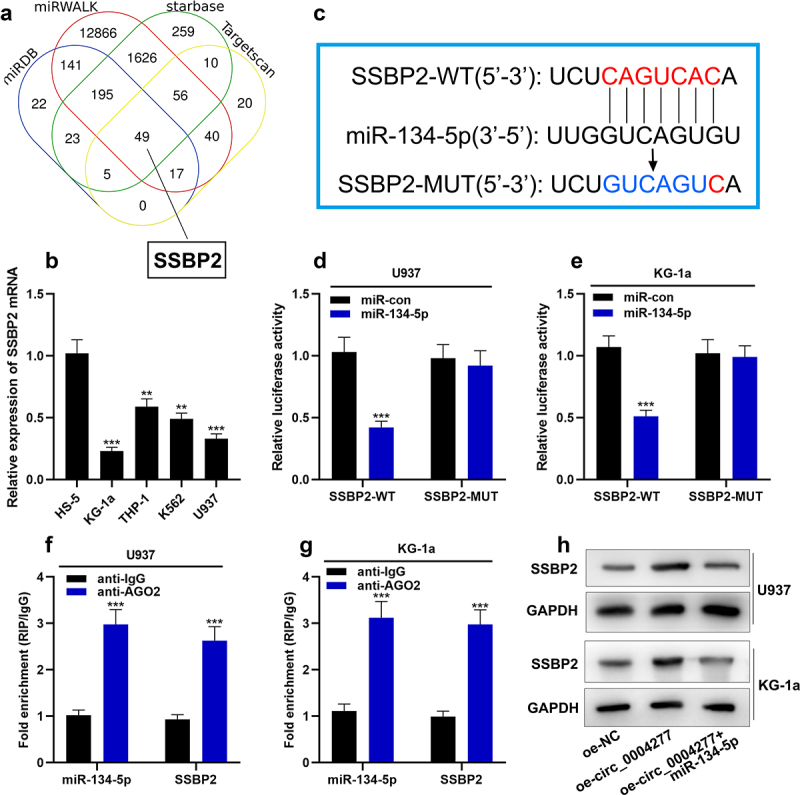
Circ_0004277 modulates SSBP2 expression in AML cells by sponging miR-134-5p A. Four online databases (Starbase, Targetscan, miRWALK, miRDB) showed that 49 mRNAs had the complementary binding sites with miR-134-5p. B. qRT-PCR revealed that SSBP2 mRNA was down-regulated in AML cells. C. Starbase dataBase showed the complementary binding sites between miR-134-5p and SSBP2 3'UTR. D-G. Dual luciferase reporter gene assay (d, e) and RIP assay (f, g) showed that miR-134-5p could bind with SSBP2 3'UTR directly. H. Western blot assay was used to measure the expression of SSBP2 after circ_0004277 and miR-134-5p were selectively regulated. ** P < 0.01, *** P < 0.001.

In order to find the downstream target of miR-134-5p in AML, four online databases (StarBase, TargetScan, miRWALK and miRDB) were used to predict the mRNAs, which have the complementary binding sites with miR-134-5p. The results showed that there were 49 mRNAs in the intersection, and SSBP2 was on our focus (Figure 6(a)). qRT-PCR showed that SSBP2 mRNA was lowly expressed in AML cells compared with HS-5 cells (Figure 6(b)). As shown in Figure 6(c), there was a complementary binding site between SSBP2 3'UTR and miR-134-5p. Dual-luciferase reporter gene assay showed that miR-134-5p substantially weakened the luciferase activity of the SSBP2-WT reporter, while it had no effect on luciferase activity in the SSBP2-MUT reporter, indicating that this binding site was functional (Figure 6(d,e)). Furthermore, the data of the RIP experiment and RNA pull-down experiment showed that SSBP2 bound with miR-134-5p in U937 and KG-1a cells (figure 6(f,g)). Western blot assay showed that circ_0004277 overexpression could up-regulate the SSBP2 expression and miR-134-5p mimics reverse this effect (Figure 6(h)).

## 4. Discussion

In recent years, circRNAs, as a novel family of non-coding RNAs, have become a hot research topic [17]. Accumulating circRNAs are found to be implicated in AML development. For example, circCRKL inhibited the proliferation and colony-forming ability of AML cells by regulating miR-196a-5p and miR-196b-5p to promote the expression of p27 [15]. Circ-foxo3 is under-expressed in AML, and it correlates with better prognosis of the patients [5]. Circ_0079480 is remarkably overexpressed in AML; knockdown of circ_0079480 restrains the growth of AML cells and stimulates their apoptosis [18]. In this work, circ_0004277, which is down-regulated in AML, is identified based on circRNA expression profiling (GSE94591) analysis. Reportedly, circ_0004277 is overexpressed in colorectal and hepatocellular carcinomas and functions as a cancer-promoting factor to promote cancer progression [19,20]. Interestingly, the present research demonstrates that circ_0004277 is under-expressed in AML. It is also observed that circ_0004277 overexpression remarkably suppresses the growth and aggressiveness of AML cells, implying that circ_0004277 is tumor-suppressive in AML.

MiRNAs can function as oncogenes or tumor suppressors in AML, modulating multiple biological processes such as growth, survival, differentiation, self-renewal, epigenetic regulation and chemoresistance [21–25]. For instance, miR-146a is a tumor suppressor that is under-expressed in myeloid leukemia; miR-146a mimics targeting myeloid cells repress nuclear factor kappa B-driven inflammatory processes and leukemia progression *in vivo* [23]. MiR-34c-5p is remarkably down-regulated in AML stem cells, and its under-expression is closely correlated with unfavorable prognosis and unfavorable treatment response in AML patients; in addition, miR-34c-5p overexpression induces senescence of AML stem cells in immunodeficient mice *in vitro*, prevents leukemogenesis and enhances eradication of AML stem cells [24]. Another study reports that miR-12462 overexpression promotes the sensitivity of AML cells to cytarabine chemotherapy and restrains the development of tumors in xenograft mice [25]. In this work, we confirm the targeting relationship between miR-134-5p and circ_0004277. Reportedly, miR-134-5p shows tumor-suppressive properties in gastric cancer and osteosarcoma [26–28]. In the research, we observe that miR-134-5p is overexpressed in AML. Inhibition of miR-134-5p markedly suppresses the growth and aggressiveness of AML cells, implying that miR-134-5p is an oncogenic miRNA in AML.

Reportedly, circRNAs work as miRNA sponges to participate in diverse biological processes [29]. Some circRNAs have been identified as competitive endogenous RNAs (ceRNAs) in AML. For instance, circ_0000488 enhances AML cell growth and impedes apoptosis by impeding miR-496 expression and enhancing protein kinase cAMP-activated catalytic subunit beta expression [30]. Circ_0004136 facilitates the progression of AML by adsorbing miR-142 [31]. Circ_0079480 is overexpressed in AML, and it facilitates the progression of AML via miR-654-3p/heparin binding growth factor axis [18]. In this work, we confirm that miR-134-5p is negatively correlated with circ_0004277 expression in bone marrow samples of AML patients, and up-regulation of miR-134-5p abolishes the repressing impact of circ_0004277 overexpression on the malignant phenotype of AML cell, suggesting that circ_0004277 is implicated in the modulation of AML development through sponging miR-134-5p.

SSBP2 is involved in interacting with single-stranded DNA, mediating DNA damage response and maintenance of genome stability. Its dysregulation is closely associated with tumorigenesis. For example, SSBP2 decreased the colony-forming ability of esophageal squamous cell carcinoma cells through inhibiting Wnt signaling pathway [32]. In addition, SSBP2 is frequently deleted in AML, and SSBP2 restoration leads to loss of clonogenicity ability and cell cycle arrest of AML cell line [33]. In our study, we demonstrate that SSBP2 is down-regulated in AML and plays as a downstream target of miR-134-5p in AML cells. Additionally, circ_0004277 can up-regulate the expression of SSBP2 by miR-134-5p. These data partly explain the mechanism of SSBP2 underexpression in AML cells.

## 5. Conclusion

In summary, this research unveils that circ_0004277 is under-expressed in AML. Circ_0004277 overexpression impedes the growth and aggressiveness of AML cells. Mechanistically, circ_0004277 upregulate SSBP2 expression by sponging miR-134-5p. This work provides innovative and important insights into the biological function of circRNA in mediating AML development and delivers some clues for the diagnosis and treatment of AML.

## Highlights

- circ_0004277 was down-regulated in AML.
- circ_0004277 overexpression remarkably repressed AML cell viability, migration and invasion.
- miR-134-5p served as a direct downstream target of circ_0004277 and SSBP2 is a target miR-134-5p.
